# Optimization of Operational Parameters of Plant Protection UAV

**DOI:** 10.3390/s24165132

**Published:** 2024-08-08

**Authors:** Wei Xing, Yukang Cui, Xinghao Wang, Jun Shen

**Affiliations:** 1 College of Automation Engineering, Nanjing University of Aeronautics and Astronautics, Nanjing 211106, China; xingwei@nuaa.edu.cn (W.X.); wangxinghao@nuaa.edu.cn (X.W.); junshen@nuaa.edu.cn (J.S.); 2 College of Mechatronics and Control Engineering, Shenzhen University, Shenzhen 518060, China

**Keywords:** plant protection, unmanned aerial vehicles, nozzle flow rate, flight speed, flight height, average deposition rate

## Abstract

The operational parameters of plant protection unmanned aerial vehicles (UAVs) significantly impact spraying effectiveness, but the underlying mechanism remains unclear. This paper conducted a full factorial experiment with varying flight speeds, heights, and nozzle flow rates to collect parameter space data. Using the Kriging surrogate model, we characterized this parameter space and subsequently optimized the average deposition rate and coefficient of variation by employing a variable crossover (mutation) probability multi-objective genetic algorithm. In the obtained Pareto front, the average sedimentation rate is no less than 46%, with a maximum of 56.08%, and the CV coefficient is no more than 13.91%, with a minimum of only 8.42%. These optimized parameters enhance both the average deposition rate and spraying uniformity compared to experimental data. By employing these optimized parameters in practical applications, a balance between the maximum average deposition rate and minimum coefficient of variation can be achieved during UAV spraying, thereby reducing pesticide usage, promoting sustainable agriculture, and mitigating instances of missed spraying and re-spraying.

## 1. Introduction

In recent years, the integration of unmanned aerial vehicles (UAVs) into agricultural practices has revolutionized traditional farming methods [1]. Among the various applications of UAVs in agriculture, the use of plant protection UAVs has garnered significant attention due to their potential to enhance crop yields, reduce pesticide usage, and mitigate environmental impacts. Plant protection UAVs offer a versatile and efficient means of applying pesticides, fertilizers, and other agrochemicals to agricultural fields with precision and a minimal environmental footprint [2,3,4].

With the advancement of aviation technology, UAV technology has matured significantly. As a novel plant protection technology, the UAV industrial system for plant protection has witnessed remarkable progress [5]. Compared to traditional spraying equipment, plant protection UAVs offer enhanced safety, efficiency, cost-effectiveness, adaptability, and efficacy in prevention and control [6,7,8]. However, challenges persist in the practical application of plant protection UAVs, including issues such as over-spraying, missed spraying, and droplet drift, leading to liquid wastage, suboptimal spraying effects, and environmental pollution [9]. Various factors influence the spraying effect of plant protection UAVs, including the aircraft type, flying height, flying speed, nozzle type, and nozzle flow rate, all of which impact the final spraying outcome [10,11,12,13]. Understanding these factors and establishing mathematical models for parameter selection are crucial for improving spraying efficiency and reducing wastage.

When employing UAVs for plant protection, the flight speed, flight height, and nozzle flow rate directly influence the deposition distribution characteristics of the spray, thereby affecting the final spraying efficacy. Hence, the operational parameters of the UAVs are pivotal in determining operational effectiveness [14,15,16]. The findings indicate that the flight speed, flight height, and nozzle flow rate of the plant protection UAVs have a descending order of impact on the spraying effect, with the flight height exerting the most significant influence, followed by the flight speed and nozzle flow rate. Fiaz Ahmad and colleagues [17,18,19] investigated the spraying effects of various combinations of flight speed, flight height, and nozzle flow rate, including the deposition rate, coverage rate, and coefficient of variation (CV), and identified the optimal parameter combination from experimental data. However, these studies solely selected the best parameter combination from obtained experimental data without establishing a mathematical model based on the data, potentially overlooking the actual best parameter combination. Lian and others [20] developed mathematical models of the operational parameters and spraying effects based on experimental data using methods such as response surface analysis and nonlinear regression derived from field experiments. They utilized these models to optimize the operational parameter combination and obtain the optimal parameter combination across the entire parameter space. Nonetheless, these modeling methods have limitations; for instance, the response surface method may not accurately represent all sample points, leading to errors, and nonlinear regression is sensitive to the initial values, potentially resulting in an inaccurate model. The Kriging model, a precise interpolation model proposed by Krige in 1973 and further refined by Matheron, overcomes these challenges [12]. It provides highly reliable predictions and standard deviation information, yielding comprehensive parameter space information at a lower experimental cost, thus ensuring accuracy in subsequent surrogate model applications and optimization results. Leveraging the advantages of accuracy and low data sample requirements of the Kriging model, this paper applies the Kriging model to complete the modeling of the objective function.

Inspired by prior research, this paper conducted a three-factor, three-level full factorial experiment covering the nozzle flow rate, flight speed, and flight height to gather data samples for parameter space modeling. The study then employed the Kriging model to characterize the relationship between the operational parameters and the spraying effects of plant protection UAVs. To address the challenges of optimizing these parameters, the study applied an enhanced multi-objective genetic algorithm (NSGA-II), specifically designed to tackle the multi-objective optimization problem of maximizing the average deposition rate and achieving uniformity (minimizing the coefficient of variation).

The key innovations of this study are as follows: This study addresses the complex interactions of UAV operational parameters and their impact on spraying effectiveness through a surrogate modeling approach, specifically using the Kriging model to map the relationships between these parameters and their effects. The primary objective is to simultaneously maximize the average deposition rate and minimize the coefficient of variation, establishing a dual-objective optimization problem. To navigate the complex parameter space characterized by multiple peaks and valleys, a variable crossover (mutation) probability strategy is integrated into the multi-objective genetic algorithm, helping to avoid local optima and find more globally optimal solutions. The post-optimization results show a maximum average deposition rate of 56.08% and a coefficient of variation of 13.91%, significantly surpassing the initial experimental results. By applying these optimized parameters, this study not only reduces pesticide use but also enhances agricultural sustainability. The optimized spraying operations improve uniformity and reduce the need for re-spraying, increasing both the efficiency and effectiveness of plant protection.

The structure of the remainder of this paper is as follows: Section 2 introduces the experimental design and process; Section 3 presents the experimental results and provides an analysis of these results; Section 4 establishes the Kriging model and utilizes an enhanced NSGA-II to optimize the operational parameters; and Section 5 presents the conclusions of this paper.

## 2. Materials and Methods

This section introduces the experimental materials and equipment required for data acquisition. This study focused on the MG-1P RTK agricultural UAV, and a three-factor three-level full factorial experiment was designed to investigate the effects of the nozzle flow rate, flight speed, and flight height on the average deposition rate and coefficient of variation after spraying. A total of 18 sampling points were set up for the spraying experiment to collect data. Deposition rate determination was conducted using a dye elution method, and the deposition rate was calculated based on the relationship between the absorbance of the eluent and the solution concentration. After collecting all experimental data, the average deposition rate and coefficient of variation at different levels of each operational parameter were calculated, and the trends of operational parameters’ influence on spraying effectiveness were analyzed.

### 2.1. Materials and Equipment

**Figure 1:**
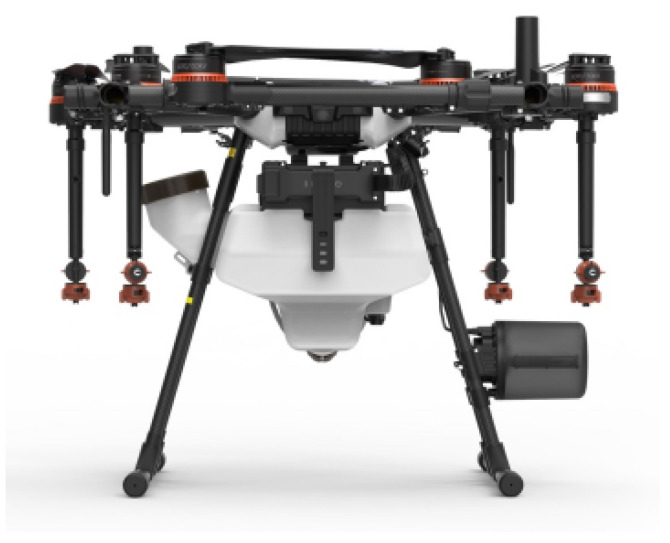
MG-1P RTK-type plant protection UAV.

The equipment selected for this experiment is the MG-1P RTK-type plant protection UAV (Figure 1) produced by Shenzhen DJI Innovations Technology Co., Ltd (Shenzhen, China). The specific parameters are shown in Table 1.

### 2.2. Experimental Design

#### 2.2.1. Sampling Point Layout

**Figure 2:**
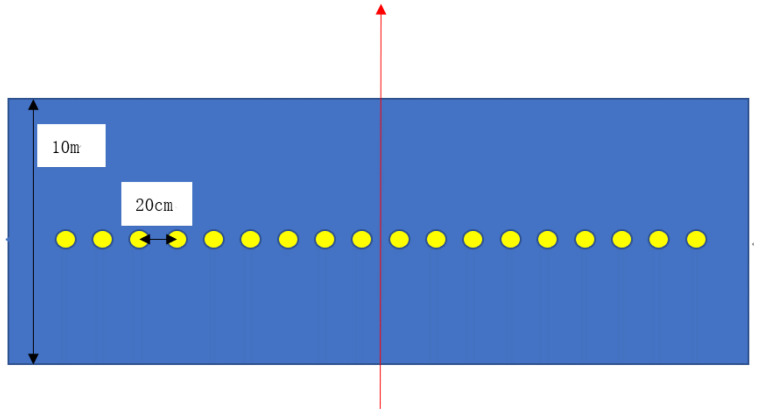
Sampling layout diagram.

**Figure 3:**
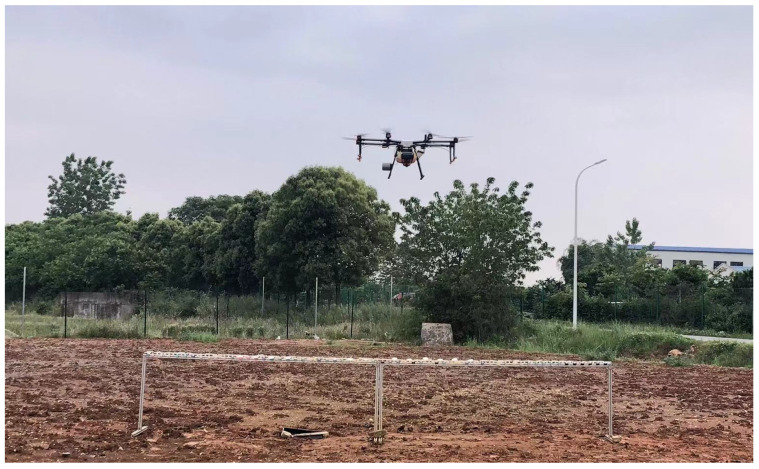
On-site sampling layout.

The experimental site was an empty area measuring 10 m in length and 20 m in width. Filter paper clips were affixed to the experimental rack at 20 cm intervals using long-tail clips, as illustrated in Figure 2. The red arrow direction within Figure 2 indicates the flight path of the UAV during operation, while the yellow circles denote the positions where filter paper was placed. This type of drone has a spraying swath of 4 m and 18 sampling points arranged with filter paper at intervals of 20 cm (without sampling at both ends). The on-site sampling layout is shown in Figure 3. The rationale for arranging sampling filter paper vertically along the flight path of the plant protection UAV is primarily based on the distribution characteristics of droplet deposition. While the amount of droplet deposition along the parallel direction of flight lines tends to be relatively uniform or similar, significant variations are observed in deposition across the perpendicular direction. This distinct distribution pattern is crucial for understanding how different operational parameters influence the uniformity of the deposition rate. By deploying the sampling papers vertically, this study aimed to capture these variations in deposition across the vertical profile of the sprayed area. This method allows for a detailed assessment of how changes in parameters such as the nozzle flow rate, flight speed, and flight height impact the evenness of the spray deposition.

The filter paper samples are positioned along the vertical flight direction to assess droplet dispersion across the effective spraying width. Prior studies indicate minimal deviation in the spraying efficacy of plant protection UAVs during parallel flight operations, contrasting sharply with the substantial variations observed in vertical flight scenarios. Consequently, sampling points are evenly distributed across the vertical width to comprehensively capture spraying performance data.

#### 2.2.2. Experimental Parameter Design

As this experiment primarily investigated the impact of various pesticide application parameters on the spraying outcomes of the plant protection UAV, the focus was on analyzing the parameters of flight speed, flight height, and nozzle flow rate. Based on the recommended operational parameters—specifically, a flight speed of 2 m/s, a flight height of 3 m, and a nozzle flow rate of 0.8 L/min—the study designed a full factorial experiment with different gradients around these values. The predetermined levels for the experimental parameters were as follows: flight speeds of 1 m/s, 2 m/s, and 3 m/s; flight heights of 2.5 m, 3 m, and 3.5 m; and nozzle flow rates of 0.6 L/min, 0.8 L/min, and 1.0 L/min.

#### 2.2.3. Test Plan Design

After transporting the plant protection UAV, sampling bracket, and other equipment to the experimental site, tools such as a tape measure were used to measure the placement point of the sampling bracket and the start and end points of the UAV operation and mark them. The selected UAV for the experiment is capable of autonomous operation, allowing for a test flight with the UAV loaded with a dye solution after marking to verify whether the flight route aligns with the predetermined route settings. In the case of any deviations in the route, the points were remarked, and the test flights were conducted until the route was accurately set. Upon the completion of each parameter group, the dye solution was allowed to dry on the filter paper for approximately 1 min. Using clean and uncontaminated tweezers, the sampling filter paper was placed in a self-sealing bag, and the sample collection bags for each group were systematically stored according to their respective numbers. Throughout the experiment, the environmental parameters were monitored. If the natural wind speed exceeded 2.5 m/s, the experiment was considered invalid, and a flight spraying experiment under the same parameters needed to be reattempted. Additionally, after every six sets of experiments, samples were extracted from the mother liquor in the UAV box to monitor the photolysis degree of the mother liquor during the experiment.

#### 2.2.4. Deposition Rate Measurement and Uniformity Calculation

After collecting the field experiment samples, the sampling filter paper must undergo elution, followed by absorbance measurement. Using a pipette, 10 mL of deionized water (to prevent interference from ions in tap water) was dispensed into the self-sealing bag. Once all samples in a group had undergone deionized water injection, the bag was shaken for 2 min until no obvious color remained on the filter paper. Subsequently, 3.5 mL of eluent was extracted from the self-sealing bag using a pipette and transferred to a colorimetric dish for measurement. Adopting the principle that absorbance increases with the lemon-yellow solution concentration at the same wavelength, this paper employed the absorbance measurement to quantify the lemon-yellow deposition amount in the sample. For the absorbance measurement, a 722S-type visible-light spectrophotometer was employed. Prior to sample absorbance measurement, the spectrophotometer required calibration, which entailed preheating the instrument for 30 min, setting the wavelength to 478 nm (the maximum absorbance wavelength of lemon yellow), placing the blank group (pure deionized water) in the spectrophotometer, closing the cover and roughly adjusting to 100%, and then opening the cover and adjusting to 0%, repeating this process until the value stabilized upon cover switching. Following spectrophotometer calibration, each set of colorimetric dishes containing samples was placed into the instrument, and their respective absorbance values were read and recorded according to group number.

Due to the photolysis characteristics of lemon-yellow dye, the absorbance–concentration calibration curve of lemon-yellow dye requires re-measurement after completing a specific experiment. This paper used an electronic balance to measure 1 g of lemon yellow, which was added to a volumetric flask to create a 2 g/L lemon-yellow mother solution by volume. This solution was subsequently diluted to form gradient standard solutions of 60 mg/L, 50 mg/L, 40 mg/L, 30 mg/L, 25 mg/L, 20 mg/L, 15 mg/L, 10 mg/L, and 5 mg/L. Table 2 shows the absorbance values of lemon-yellow solutions at different concentration gradients.

**Figure 4:**
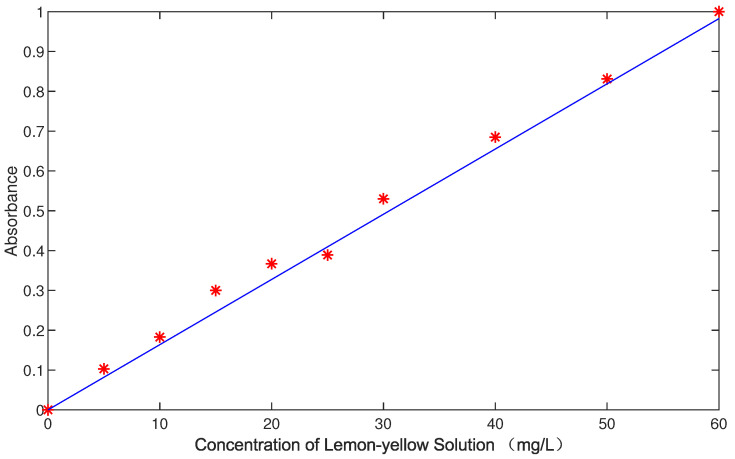
Lemon-yellow solution concentration–absorbance curve.

After obtaining the standard solution and its absorbance value, the ‘Polyfit’ function in Matlab was used to conduct linear fitting on the above data. Since the absorbance value of the lemon-yellow solution is directly proportional to its concentration, linear fitting is applicable in this case. By using the above data, a concentration–absorbance curve for the lemon-yellow solution, as depicted in Figure 4, was derived through linear fitting.

Therefore, the concentration–absorbance curve equation for the lemon-yellow solution is

(1) A=0.0164x,

where *A* is the absorbance value, and *x* is the concentration of the lemon-yellow solution. When fitting, the intercept should be 0.0212, but because this paper used pure deionized water as the calibration basis (that is, it is assumed that when the absorbance is 0, the concentration of lemon-yellow solution should also be 0), the intercept is taken as 0.

In order to demonstrate the uniformity of the deposition rate at each sampling point under different operational parameters of the plant protection UAV, this paper uses CV as the uniformity measure of the deposition rate at each sampling point under different parameters. The calculation method of the coefficient of variation, CV, is

(2) $$\mathrm{CV}=\frac{P}{\bar{X}}\times 100\%,$$

(3) $$P=\sqrt{\sum_{i=1}^{n}\frac{{\left(X_{i}-\bar{X}\right)}^{2}}{n-1}},$$

where *P* is the standard deviation of the deposition rate of each sample under this group of parameters, $X_{i}$ is the deposition rate of each sampling point, $\bar{X}$ is the average deposition rate of sampling points in this group of experiments, and *n* is the total number of sampling points in this group.

After measuring the absorbance values of each group of samples, the deposition rate values of each sample are calculated according to

(4) $$\beta =\frac{10\gamma }{kSM\varepsilon },$$

(5) $$S=\frac{\pi D^{2}}{4},$$

where 10 is the volume of the eluent (mL), γ is the absorbance value of the sample, *k* is the slope of the concentration–absorbance curve of the lemon-yellow solution, *S* is the area of filter paper (m^2^), *M* is the amount of pesticide solution under this group of experimental parameters (L/m^2^), ε is the concentration of mother liquor, and *D* is the diameter of the filter paper.

## 3. Results and Analysis

This section presents the modeling of the parameter space using the Kriging model based on the experimental data from Section 2. Following the description of the multi-objective optimization problem in this paper, a multi-objective genetic algorithm is employed to solve the optimization problem. The results obtained are not superior to the experimental data, as the algorithm is prone to getting trapped in local optima due to multiple peaks and valleys in the parameter space. To address this issue, a strategy of variable crossover (mutation) probability is implemented to improve the multi-objective genetic algorithm. The final Pareto frontier results outperform the original experimental data.

### 3.1. Results

The original experimental results calculated by (2)–(5) are shown in Table 3. The average deposition rate of the sample points in the table represents the deposition value of the final pesticide (lemon-yellow solution) relative to the total amount of spraying; that is, it represents a certain degree of the pesticide utilization rate. The coefficient of variation characterizes the uniformity of the deposition rate at each point; that is, it characterizes the degree of re-spraying and missed spraying within the entire spray range. The above two indicators are usually used as characteristics to evaluate the spraying effect of pesticide application equipment, so these two indicators are statistically calculated.

The environmental parameters for the 1–9 group data are an average natural wind speed of 1.6 m/s, an average ambient temperature of 28.4 °C, and an average relative humidity of 52.3%. The environmental parameters for the 10–18 group data are an average natural wind speed of 1.5 m/s, an average ambient temperature of 27.4 °C, and an average relative humidity of 51.6%. The environmental parameters for the 19–27 group data are an average natural wind speed of 1.5 m/s, an average ambient temperature of 29.2 °C, and an average relative humidity of 53.8%.

### 3.2. Analysis of Results

The observed decrease in the average deposition rate with an increase in the nozzle flow rate—from 42.40% at 0.6 L/min to 29.57% at 1.0 L/min—can be attributed to the dynamics of droplet formation and behavior. Higher flow rates typically produce larger droplets due to increased liquid throughput per unit time. These larger droplets possess greater momentum, which can lead to a higher impact on the target surface but with less dispersion and a smaller coverage area. The heavier droplets are less influenced by air resistance and are more prone to follow a straight trajectory, leading to increased deposition in targeted areas but reduced overall coverage. This trend suggests a trade-off between achieving high localized deposition and ensuring broad coverage.

The positive correlation between the flight speed and deposition rate—from 26.57% at 1 m/s to 40.67% at 3 m/s—highlights the effects of droplet travel time and evaporation. At higher speeds, the UAV covers more ground quickly, reducing the time during which droplets are suspended in the air. This reduction in airtime decreases the likelihood of droplet evaporation and drift, common issues at lower speeds, where droplets have more time to be affected by environmental factors like wind. Higher speeds can lead to more efficient droplet deposition on the crop canopy before the droplets can disperse or evaporate, effectively enhancing the deposition rate.

The fluctuation in deposition rate with varying flight heights—initially decreasing at 3.0 m and then increasing at 3.5 m—can be explained by the complex interplay between droplet dispersion and drift. At lower heights (2.5 m), the proximity to the target surface reduces drift significantly, improving the deposition efficiency. However, as the height increases to 3.0 m, slight increases in drift may occur, dispersing droplets over a wider area but reducing their concentration in target zones, thus lowering deposition rates. The subsequent increase in deposition rate at 3.5 m could be due to the stabilization of droplet dispersion patterns, where the balance between height and wind dynamics allows for the effective spreading of droplets over the intended area without significant loss to drift.

The CV demonstrates a nonlinear trend with an increase in the nozzle flow rate: starting at 27.05% at 0.6 L/min, rising to 36.67% at 0.8 L/min, and then slightly decreasing to 29.51% at 1.0 L/min. This initial increase can be attributed to the larger droplets formed at higher flow rates, which, while they may achieve more targeted and dense deposition, tend to distribute less evenly across a broader area. This results in a higher variability in the deposition rate across the sprayed area. The slight decrease in CV at the highest flow rate suggests that, while the droplets are larger, the sheer volume of fluid may somewhat compensate for the uneven distribution, slightly reducing variability by saturating more of the target area uniformly.

The CV decreases consistently with increasing flight speed: from 36.87% at 1 m/s to 30.30% at 2 m/s down to 26.08% at 3 m/s. This trend suggests that higher speeds contribute to a more uniform spray distribution. At higher speeds, the UAV passes quickly over the area, reducing the time for environmental factors like wind to affect the droplet path. This rapid transit ensures a more consistent application over the sprayed area, reducing the variance in deposition from one area to another. Faster speeds may also promote a more even dispersal of droplets due to the increased air turbulence generated by the UAV’s movement, aiding in a more homogeneous spread over the crops.

The CV shows a complex pattern as the height changes: starting at 33.40% at 2.5 m, peaking at 37.40% at 3.0 m, and then significantly dropping to 22.45% at 3.5 m. This suggests that intermediate heights may expose the droplets to varying wind currents that can disrupt uniform spray patterns, increasing the variability in deposition. At the lowest height, the proximity to the target reduces the impact of wind, moderately controlling the CV. The significant decrease in CV at 3.5 m could be indicative of a height where the droplets have sufficient time to disperse under controlled conditions, achieving a balance between the spread and drift and thus optimizing uniformity.

In summary, the influence of each operational parameter of the plant protection UAV on the average deposition rate and coefficient of variation differs. This discrepancy highlights the complexity of the optimization problem, suggesting the need for a multi-objective optimization approach to effectively balance conflicting objectives.

## 4. Mathematical Modeling and Parameter Optimization

### 4.1. Mathematical Modeling

This study utilized Matlab R2019a for program execution. Specifically, the ‘dacefit’ function from the DACE toolbox [21], which implements the Kriging model, was employed to model the sample data and predict the input data. The DACE toolbox provides six correlation models and three regression models, of which this paper selected the zero-order polynomial regression and Gaussian regression models. The initial theta value is set to 10, with upper and lower limits of 20 and 0.1, respectively. The selection of the zero-order polynomial regression and Gaussian correlation models in the DACE framework is strategically aimed at modeling the effects of operational parameters such as the flight speed, height, and nozzle flow rate on the deposition rate and uniformity in UAV spraying. The zero-order polynomial is used, recognizing that the outputs are influenced more by the combined spatial configuration of inputs than by individual variable effects, suitable for the complex interactions in UAV operations. The Gaussian correlation model is chosen for its ability to deliver smooth predictions, which is essential for capturing the nuanced changes in spraying outcomes with variations in UAV settings. The initial theta value is set at 10 with broad bounds, allowing the model to be finely adjusted to the sensitivity of the deposition metrics to the operational parameters, ensuring it accurately reflects the true behavior of UAV spraying without overfitting or underfitting. This configuration assists in optimizing the effectiveness and efficiency of plant protection UAVs.

**Figure 5:**
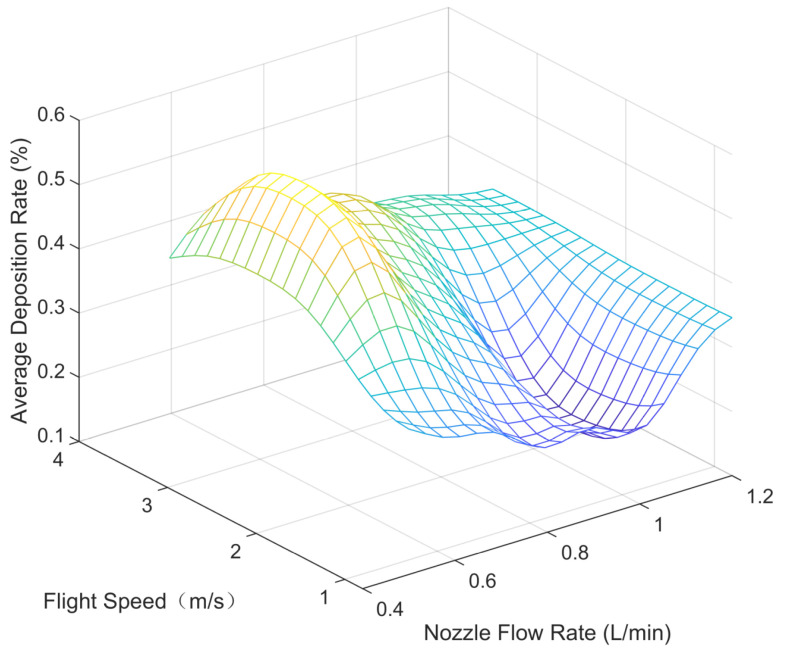
Parameter space between flight speed and nozzle flow rate and average deposition rate.

**Figure 6:**
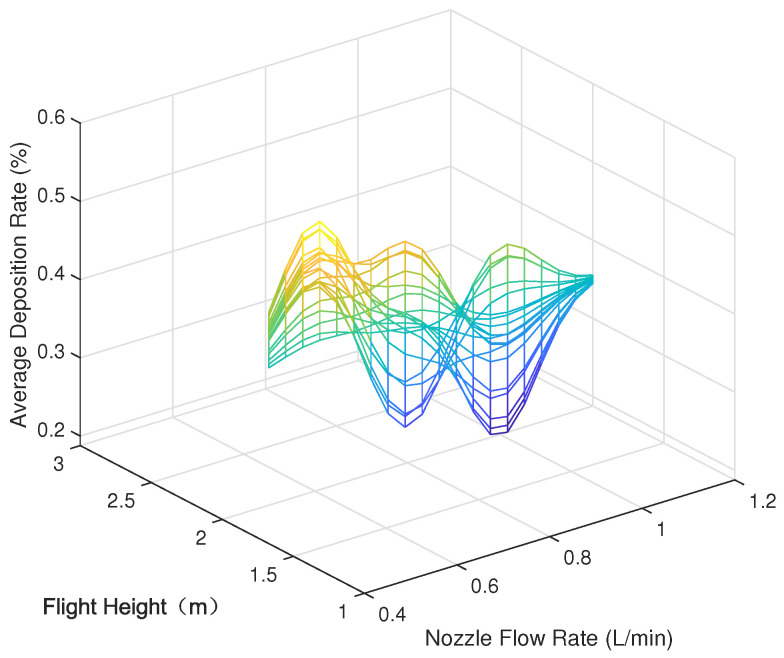
Parameter space between flight height and nozzle flow rate and average deposition rate.

**Figure 7:**
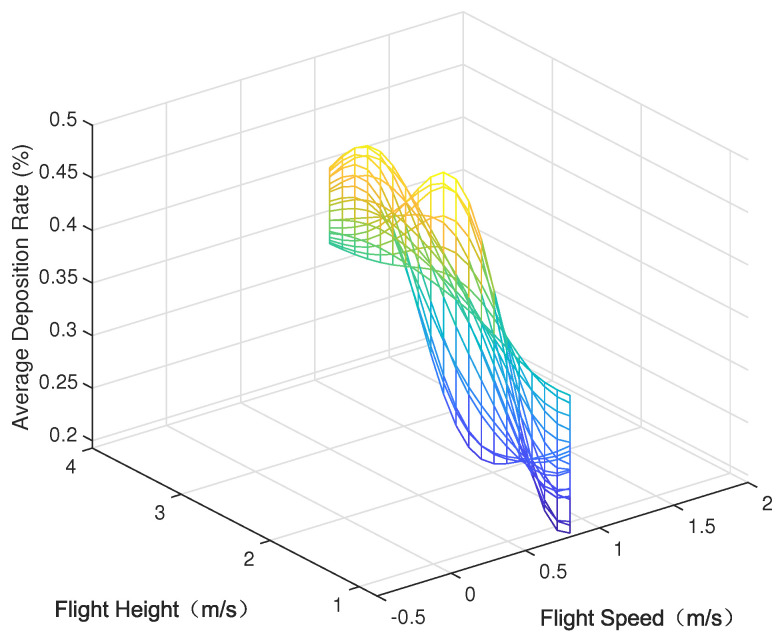
Parameter space between flight height and flight speed rate and average deposition rate.

**Figure 8:**
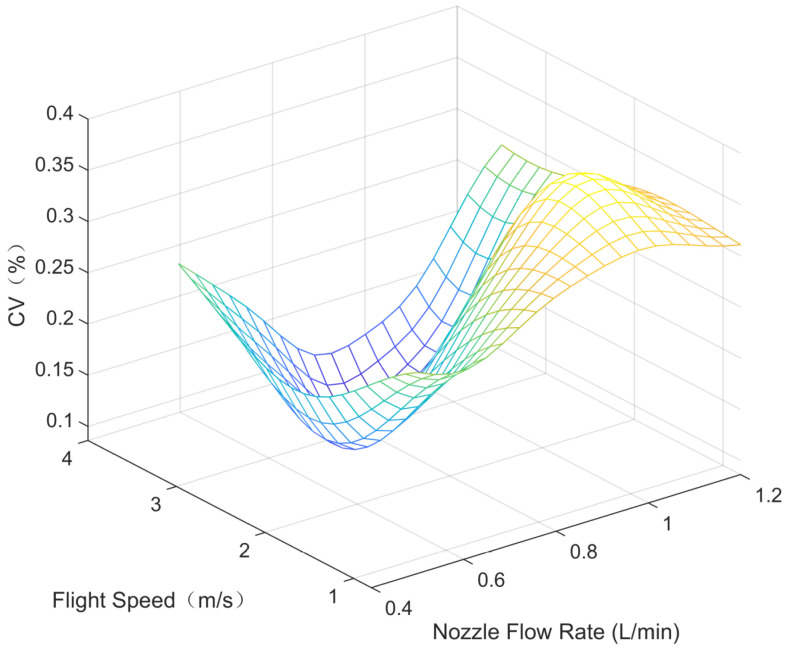
Parameter space between flight speed and nozzle flow rate and CV.

**Figure 9:**
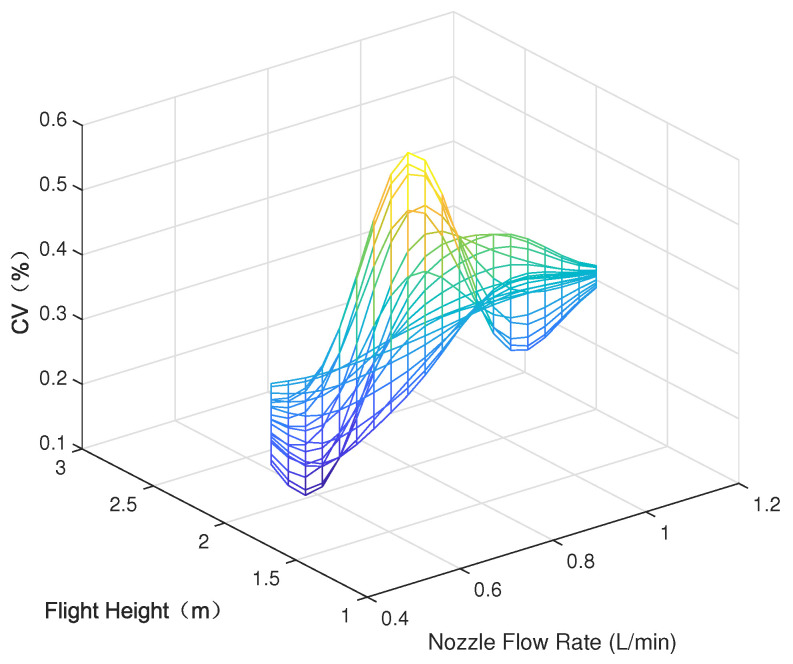
Parameter space between flight height and nozzle flow rate and CV.

**Figure 10:**
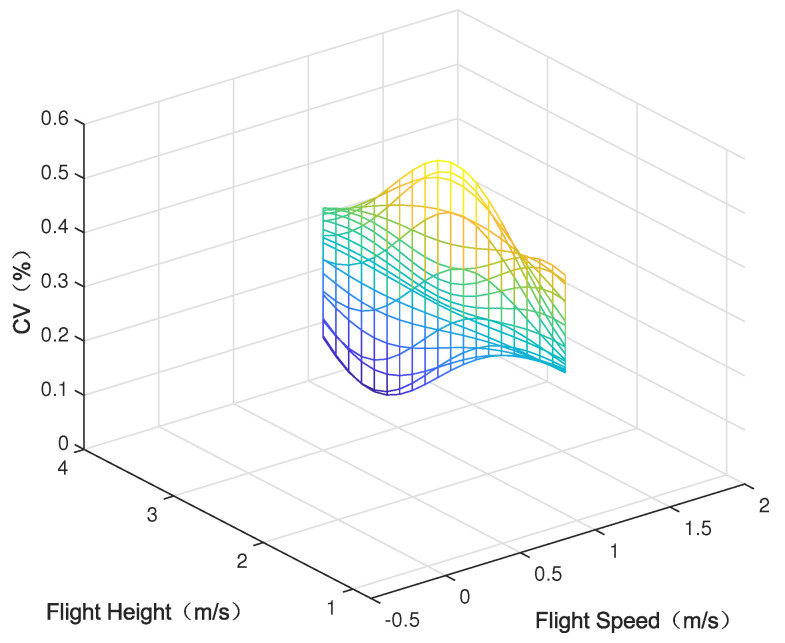
Parameter space between flight height and nozzle flow rate and CV.

Parametric modeling was conducted using the experimental data outlined in Section 3, depicting the parameter space between each variable (nozzle flow rate, flight speed, and flight height) and the modeled average deposition rate and uniformity (coefficient of variation), as shown in Figure 5, Figure 6, Figure 7, Figure 8, Figure 9 and Figure 10.

In the figures, the flight height is held constant at 3.5 m while analyzing the parameter space between the nozzle flow rate and flight speed and the average deposition rate or uniformity. Similarly, the flight velocity is fixed at 2 m/s when examining the parameter space between the nozzle flow rate and flight height and the average deposition rate or uniformity. Lastly, the nozzle flow rate is maintained at 0.8 L/min when exploring the parameter space between flight speed and flight height versus the average deposition rate or uniformity.

Figure 5 displays the distribution of the average deposition rate at different flight speeds and nozzle flow rates at a fixed flight height of 3.5 m. Figure 6 illustrates the average deposition rate distribution at different flight speeds and nozzle flow rates at a constant flight speed of 2 m/s. Figure 7 depicts the average deposition rate distribution at different flight speeds and flight heights with the nozzle flow rate set to 0.8 L/min. Figure 8, Figure 9 and Figure 10 show the uniformity distribution for different combinations of flight speed, flight height, and nozzle flow rate, as described above.

### 4.2. Validation of the Fitted Kriging Model

**Figure 11:**
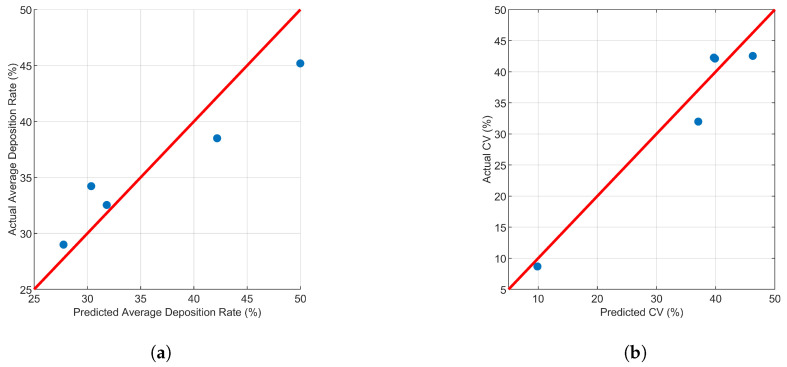
Comparison of predicted values and actual values. (**a**) Average deposition rate. (**b**) CV.

In order to verify the accuracy of the Kriging model, five randomly selected experiments were conducted to detect the relative error between the predicted and actual values of the model. The results of the five experiments are shown in Table 4, and the operational parameter combinations of these five experiments were input into the established Kriging model to obtain estimated values. From the relative errors of each parameter group, the maximum relative prediction error of the model for the average deposition rate does not exceed 13%, averaging 7.56%, while the maximum relative prediction error for CV does not exceed 16%, averaging 9.97%. By comparison, as shown in Figure 11, the predicted values provided by the Kriging model have a small difference from the actual values. Therefore, predicting the spraying effect through the established Kriging model is feasible and reliable.

### 4.3. Multi-Objective Optimization

The purpose of this optimization problem is to obtain the optimal spraying effect by selecting the appropriate operational parameters for the plant protection UAV, that is, the maximum average deposition rate and the best uniformity (minimum coefficient of variation). Therefore, this bi-objective optimization problem can be described as follows:

- Object:
- Maximum average deposition rate
- Minimum CV
- Subject to:
- 0.5 L/min ≤ nozzle flow rate ≤ 1.2 L/min
- 0.8 m/s ≤ flight speed ≤ 3.5 m/s
- 2 m ≤ flight height ≤ 4 m

During the optimization process employing the multi-objective genetic algorithm, the fitness value, which represents the objective function, is predicted by the established Kriging model. The parameters of the multi-objective genetic algorithm are outlined in Table 5. The NSGA-II (Non-dominated Sorting Genetic Algorithm II) [22] is utilized to optimize the operational parameters of the UAV for plant protection. This choice is driven by NSGA-II’s effectiveness in managing complex multi-objective optimization scenarios, which are typical in balancing the deposition rate and the CV in pesticide-spraying tasks. The algorithm is configured with a population size of 50 to balance computational efficiency while maintaining a diverse genetic pool, which is essential for a comprehensive exploration of potential solutions. The algorithm proceeds through 500 iterations, allowing for extensive evolution and refinement of solutions toward an optimal Pareto front.

A high crossover probability of 0.8 is selected to promote the exploration of new genetic combinations, enhancing the potential for discovering superior solutions, while a mutation probability of 0.2 helps maintain genetic diversity within the population, preventing premature convergence to local optima. The fitness function, based on non-dominated sorting, enables the efficient evaluation of the trade-offs between objectives, facilitating the systematic navigation of the UAV’s operational parameters—flight speed, height, and nozzle flow rate. This approach leads to optimized solutions that significantly improve both the effectiveness and efficiency of UAV pesticide application, supporting sustainable agricultural practices with reduced environmental impact. Through the strategic application of NSGA-II, this study demonstrates significant advancements in precision agriculture technologies by optimizing the variability in deposition, thus optimizing both the effectiveness and efficiency of the pesticide application.

**Figure 12:**
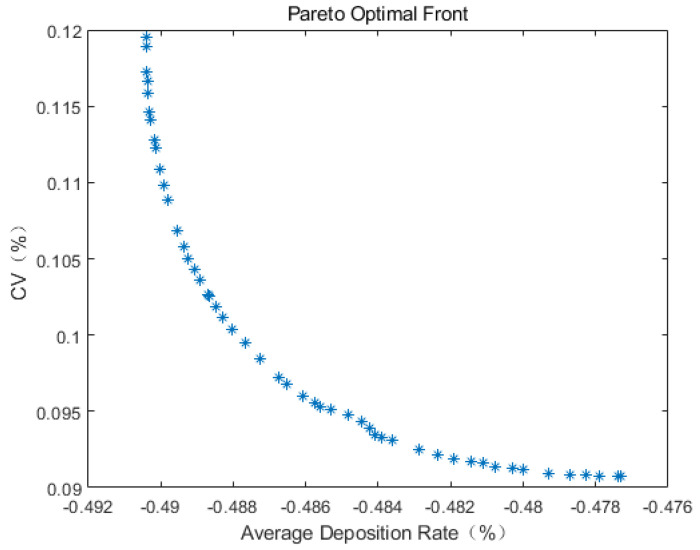
Pareto front of average deposition rate−coefficient of variation.

Figure 12 illustrates the Pareto frontier of the average deposition rate–coefficient of variation following optimization with the multi-objective genetic algorithm. Each point on the graph represents a specific optimal solution, which can guide the selection of the corresponding parameters for plant protection UAV spraying operations during practical production.

The multi-objective genetic algorithm employed in this study is aimed at minimization, with the objectives being the maximum average deposition rate and the minimum coefficient of variation. Therefore, after obtaining the predicted objective function values (average deposition rate and CV), the predicted average deposition rate is transformed to its opposite value before non-dominated sorting. Thus, the average deposition rate shown in the figure represents the opposite value of the actual average deposition rate.

From Figure 12, it can be observed that the optimization effect on the average deposition rate after optimization is not prominent and even lower than the optimal value of the average deposition rate obtained from the samples, while the optimization effect on the coefficient of variation is significant. Considering the presence of multiple peaks and valleys in each parameter space model in Figure 4, it is concluded that the diversity of solutions in the multi-objective genetic algorithm process is insufficient.

**Figure 13:**
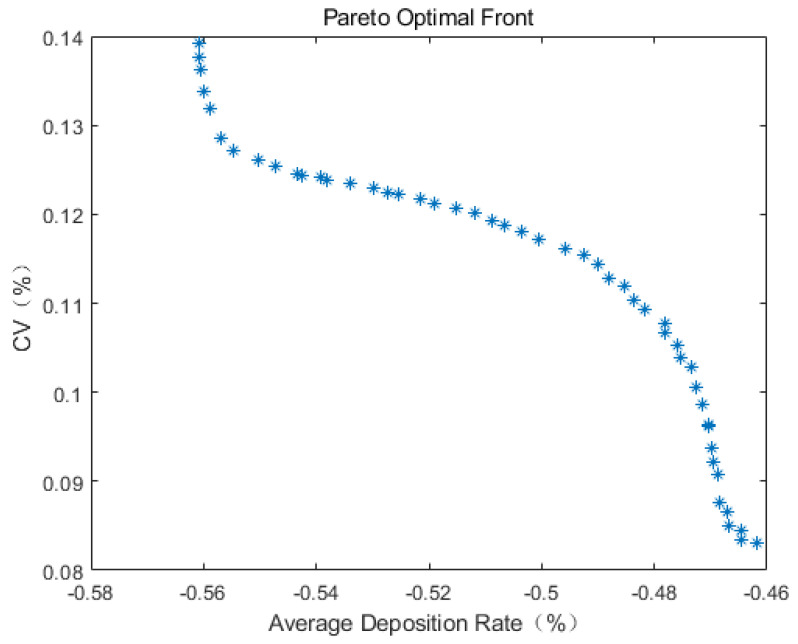
Pareto front of average deposition rate−coefficient of variation after algorithm improvement.

To address the issue of insufficient solution diversity in the multi-objective genetic algorithm optimization process, preventing the algorithm from converging to local optima, this paper adopts a variable crossover (mutation) probability strategy. This strategy ensures sufficient crossover probability in the early stages of algorithm optimization and increases the mutation probability in the later stages. The rationale behind this strategy is as follows: the crossover operation primarily utilizes parental information to generate offspring and cannot produce new genotypes. If the parent itself represents a local optimal solution, the offspring will predominantly inherit the local optimal solution information from the parent, resulting in a low probability of finding other solutions in the parameter space. Mutation operations can assist offspring in escaping local optimal solutions and enhance the ability to discover global optimal solutions. This strategy promotes the extensive exploration of the solution space through a high crossover probability in the early stages while intensifying the precision of local searches in later stages by increasing the mutation probability. This effectively balances the needs for exploration and exploitation, enhancing the algorithm’s ability to find global optima in complex multi-objective settings. The dynamic adjustment mechanism makes the algorithm better suited to handle variable and intricate optimization challenges, effectively preventing premature convergence to local optima. The variable crossover (mutation) probability strategy is implemented by

(6) $$c_{1}=\frac{T-t}{T},c_{1}+c_{2}=1,$$

where $c_{1}$ is the crossover probability, and $c_{2}$ is the mutation probability. *T* is the total number of iterations, and *t* is the current iteration number. Using the improved multi-objective genetic algorithm to optimize the above multi-objective optimization problem again results in the Pareto frontier shown in Figure 13.

The information of the top fifteen individuals in the optimization results is shown in Table 6. It can be seen from the information in the table that after optimizing the operational parameters of the plant protection UAV, the maximum average deposition rate can reach 56.08%, and the corresponding coefficient of variation is 13.91%, compared with the maximum deposition rate of 54.23% and the corresponding coefficient of variation of 17.18% in the test sample. The average deposition rate and the uniformity of the spraying effect can be improved to a certain extent by using a nozzle flow rate of 0.60 L/min, a flight speed of 2.59 m/s, and a flight height of 3.52 m as the operational parameters for plant protection UAV spraying operations. By evaluating its mean square error in the Kriging model, the average predicted mean square error of the above parameters is 0.0032, which is close to 0, so the parameter is considered to conform to the actual situation and can be used to guide actual production.

## 5. Conclusions

The operational parameters of plant protection UAV spraying have a significant impact on the effectiveness of such operations. This study has focused on the nozzle flow rate, flight speed, and flight height of the plant protection UAV.

To establish the relationship between the operational parameters of the plant protection UAV and the average deposition rate and uniformity after spraying, a three-factor three-level full factorial experiment was conducted. This experiment involved varying the nozzle flow rate (0.6 L/min, 0.8 L/min, 1.0 L/min), flight speed (1.0 m/s, 2.0 m/s, 3.0 m/s), and flight height (2.5 m, 3.0 m, 3.5 m) to collect data. Delineating the correlation between the operational parameters of plant protection UAV spraying and the average deposition rate and uniformity after spraying, this paper has utilized the collected data combined with the Kriging model to establish the plant protection UAV’s operational parameter–deposition rate (uniformity) model.

Given the complex nature of the parameter space, characterized by multiple peaks and valleys, a variable crossover (mutation) probability multi-objective genetic algorithm is proposed to address the multi-objective optimization problem. The multi-objective optimization results show a maximum average deposition rate of 56.08% with a corresponding coefficient of variation of 13.91%. Compared to experimental data, the optimized operational parameters have yielded better average deposition rates and spraying uniformity.

Applying these optimized parameters in practice can achieve a balance between the maximum deposition rate and minimum coefficient of variation, leading to reduced pesticide usage, environmentally friendly agriculture, higher deposition rates, uniform spraying, and fewer instances of missed spraying and re-spraying.

## Figures and Tables

**Table 1:** Parameters of plant protection UAV.

Main Parameter	Norms and Numerical Value
Type	MG-1P RTK
Unfold fuselage size/mm × mm × mm	1460 × 1460 × 578
Maximum load/L	10
Maximum operating flight speed/(m/s)	7
Maximum take-off altitude/m	2000
Flight duration (full load)/min	20
Maximum route duration/km	3
Minimum safe flight altitude/m	1.5
Spraying swath/m	4

**Table 2:** Absorbance value of tartrazine solution at standard concentration.

Concentrations of Lemon-Yellow Solution (mg/L)	Absorbance Values
60	1.030
50	0.831
40	0.685
30	0.530
25	0.389
20	0.367
15	0.300
10	0.183
5	0.103
0	0.000

**Table 3:** Sample data results.

Number	Nozzle Flow Rate (L/min)	Flight Speed (m/s)	Flight Height (m)	Average Deposition Rate (%)	CV (%)
1	0.6	1	2.5	39.92	26.05
2	0.6	1	3.0	34.99	47.29
3	0.6	1	3.5	30.11	25.23
4	0.6	2	2.5	48.82	10.32
5	0.6	2	3.0	45.09	28.92
6	0.6	2	3.5	53.14	14.04
7	0.6	3	2.5	43.61	43.58
8	0.6	3	3.0	31.65	30.85
9	0.6	3	3.5	54.23	17.18
10	0.8	1	2.5	19.67	49.24
11	0.8	1	3.0	26.34	40.01
12	0.8	1	3.5	22.42	31.30
13	0.8	2	2.5	46.31	38.70
14	0.8	2	3.0	23.63	58.41
15	0.8	2	3.5	36.25	22.66
16	0.8	3	2.5	39.42	39.67
17	0.8	3	3.0	42.15	41.68
18	0.8	3	3.5	46.55	8.53
19	1.0	1	2.5	22.15	30.52
20	1.0	1	3.0	24.35	47.54
21	1.0	1	3.5	19.18	34.67
22	1.0	2	2.5	30.87	41.62
23	1.0	2	3.0	42.36	24.37
24	1.0	2	3.5	18.83	33.66
25	1.0	3	2.5	40.32	20.90
26	1.0	3	3.0	29.88	17.49
27	1.0	3	3.5	40.32	14.82

**Table 4:** The accuracy of the established Kriging model.

Operational Parameters ([Nozzle Flow Rate, Flight Speed, Flight Height])	Predicted Average Deposition Rate (%)	Actual Average Deposition Rate (%)	Relative Error (%)	Predicted CV (%)	Actual CV (%)	Relative Error (%)
[0.7,1.5,3.0]	32.54	31.82	2.26	46.27	42.54	8.77
[0.8,2.8,3.6]	45.19	49.99	9.60	9.88	8.68	13.82
[0.9,1.8,3.2]	29.00	27.76	4.47	39.70	42.27	6.08
[0.7,2.4,2.8]	38.50	42.18	8.72	39.88	42.11	5.30
[1.0,2.3,2.5]	34.22	30.36	12.71	37.06	31.98	15.88

**Table 5:** Parameter settings of NSGA-II.

Parameter	Value
Population size	50
Number of iterations	500
Crossover probability	0.8
Mutation probability	0.2
Fitness function	Non-dominated sorting

**Table 6:** Optimization result information.

Nozzle Flow Rate (L/min)	Flight Speed (m/s)	Flight Height (m)	Average Deposition Rate (%)	CV (%)
0.60	2.59	3.52	56.08	13.91
0.63	2.40	3.52	55.08	12.61
0.73	2.86	3.53	47.55	10.43
0.63	2.44	3.52	55.73	12.93
0.69	2.73	3.53	50.00	11.71
0.66	2.62	3.53	52.27	12.22
0.64	2.45	3.53	53.86	12.46
0.61	2.59	3.52	56.07	13.80
0.79	2.98	3.53	46.58	8.42
0.61	2.44	3.49	55.88	13.07
0.63	2.48	3.50	55.33	12.37
0.60	2.47	3.51	56.00	13.44
0.69	2.79	3.52	49.48	11.57
0.65	2.52	3.51	53.53	12.37

## Data Availability

The original contributions presented in the study are included in the article. Further inquiries can be directed to the corresponding author.
